# A Follicle Rupture Assay Reveals an Essential Role for Follicular Adrenergic Signaling in *Drosophila* Ovulation

**DOI:** 10.1371/journal.pgen.1005604

**Published:** 2015-10-16

**Authors:** Lylah D. Deady, Jianjun Sun

**Affiliations:** 1 Department of Physiology and Neurobiology, University of Connecticut, Storrs, Connecticut, United States of America; 2 Institute for Systems Genomics, University of Connecticut, Storrs, Connecticut, United States of America; Cornell University, UNITED STATES

## Abstract

Ovulation is essential for the propagation of the species and involves a proteolytic degradation of the follicle wall for the release of the fertilizable oocyte. However, the precise mechanisms for regulating these proteolytic events are largely unknown. Work from our lab and others have shown that there are several parallels between *Drosophila* and mammalian ovulation at both the cellular and molecular levels. During ovulation in *Drosophila*, posterior follicle cells surrounding a mature oocyte are selectively degraded and the residual follicle cells remain in the ovary to form a corpus luteum after follicle rupture. Like in mammals, this rupturing process also depends on matrix metalloproteinase 2 (Mmp2) activity localized at the posterior end of mature follicles, where oocytes exit. In the present study, we show that Mmp2 activity is regulated by the octopaminergic signaling in mature follicle cells. Exogenous octopamine (OA; equivalent to norepinephrine, NE) is sufficient to induce follicle rupture when isolated mature follicles are cultured *ex vivo*, in the absence of the oviduct or ovarian muscle sheath. Knocking down the alpha-like adrenergic receptor Oamb (Octoampine receptor in mushroom bodies) in mature follicle cells prevents OA-induced follicle rupture *ex vivo* and ovulation *in vivo*. We also show that follicular OA-Oamb signaling induces Mmp2 enzymatic activation but not Mmp2 protein expression, likely via intracellular Ca^2+^ as the second messenger. Our work develops a novel *ex vivo* follicle rupture assay and demonstrates the role for follicular adrenergic signaling in Mmp2 activation and ovulation in *Drosophila*, which is likely conserved in other species.

## Author Summary

Ovulation is the process of releasing fertilizable oocytes from the ovary and is essential for metazoan reproduction. Our recent work has demonstrated principles governing ovulation process that are highly conserved across species, such that both mammals and *Drosophila* utilize matrix metalloproteinase (Mmp) to degrade extracellular matrix and weaken the follicle wall for follicle rupture. However, a fundamental question remaining in the field is how Mmp activity is precisely regulated during ovulation. This paper reports that *Drosophila* octopamine (OA), the insect equivalent of norepinephrine (NE), is the signal to induce Mmp activity through activating its receptor Oamb on mature follicle cells and that this may induce ovulation. These findings allow us to develop the first *ex vivo* follicle rupture assay for *Drosophila*, which gives us unprecedented ability to characterize the entire follicle rupturing process *ex vivo* and to identify essential factors for ovulation. Furthermore, we show that NE partially fulfills OA’s role in inducing follicle rupture *ex vivo*, indicating that follicular adrenergic signal is a conserved signal to regulating Mmp activity and ovulation. Our work not only sheds light on the long-standing question of Mmp regulation, but also may lead to a better understanding of Mmp and NE linked pathological processes including cancer metastasis and polycystic ovary syndrome.

## Introduction

Ovaries in organisms ranging from humans to insects are extensively innervated [1–4], and neuronal inputs likely play important roles in ovarian physiology [5]. In mammals, ovaries are predominantly innervated by sympathetic fibers from the ovarian plexus nerve and the superior ovarian nerve [6], which release norepinephrine (NE) locally and contribute to follicle development [7]. Deregulation of sympathetic nerve outflow to ovaries is associated with polycystic ovary syndrome (PCOS), a common endocrine disorder leading to anovulatory infertility [8,9]. Despite the apparent importance of sympathetic innervation, however, it is not yet clear how the neuronal modulators/transmitters released from nerve termini affect ovulation [10–16].

**Fig 1:**
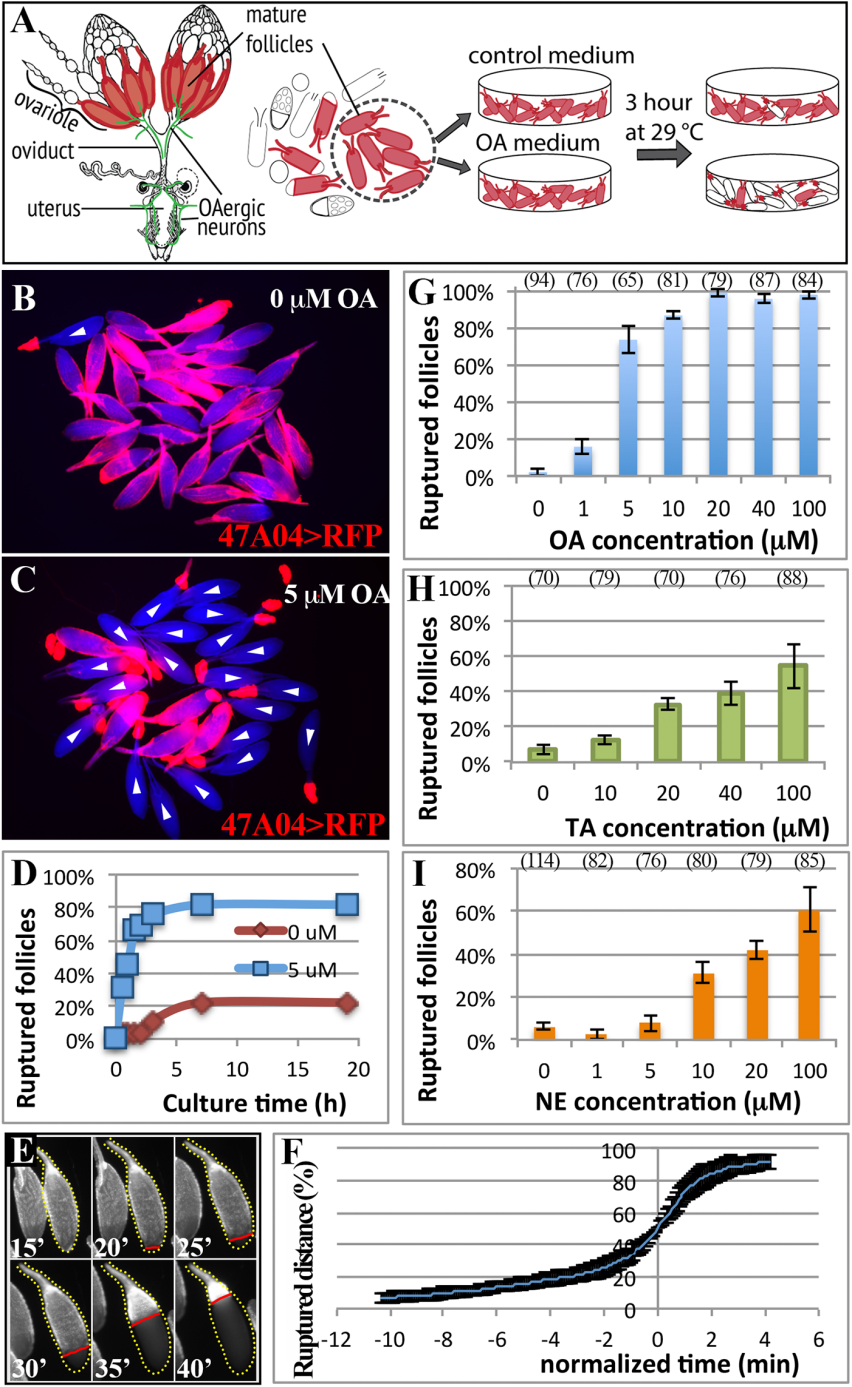
A novel *ex vivo* follicle rupture assay in *Drosophila*. (A) A schematic diagram representing the female reproductive system and *ex vivo* experiments. Mature follicle cells are marked by fluorescent proteins (red), and octopaminergic neurons are shown in green [3]. (B-C) Representative images show mature follicles after three-hour culture without (B) or with (C) OA. Follicles are imaged with incident light shown in blue and follicle cells are marked by *R47A04-Gal4* driving *UAS-RFP* (*47A04>RFP*) expression in red. White arrowheads indicate ruptured follicles here and in subsequent figures. (D) The cumulative percentage of ruptured follicles throughout the 19-hour culture period. Twenty-seven and 50 mature follicles were used in the control (0 μM) and experimental (5 μM) group, respectively. (E) A time-lapse image shows the entire follicle rupturing process after 20 μM of OA stimulation. The dotted yellow line outlines the rupturing follicle and the straight red line marks the posterior leading edge of the follicle-cell layer. Time is in minutes. (F) The kinetics of the rupturing process is similar between follicles. Data were pooled from two independent experiments, and nine out of 28 follicles isolated from ten females were analyzed. (G-I) Percentage of ruptured follicles after three-hour culture with different concentrations of OA (G), TA (H), and NE (I). Errors are standard deviations. The number of follicles analyzed was in the parenthesis above the charts in this figure and all the following figures. All conditions have three replicates except for 0 μM NE, which has four replicates.

In *Drosophila* and other insects, the biogenic monoamines tyramine (TA) and octopamine (OA) act as functional counterparts to mammalian epinephrine and norepinephrine and regulate a variety of behaviors, including the fight-or-flight response, motivation, aggression, and reproduction [17,18]. Analogous to the adrenergic innervation in mammalian ovaries, *Drosophila* octopaminergic neurons innervate ovaries and the female reproductive tract (Fig 1A; [3,19,4]). OA released from these neurons is essential for ovulation, as mutations that disrupt the enzymes required for OA synthesis, tyrosine decarboxylase 2 (Tdc2) and tyramine β-hydroxylase (TβH), completely block ovulation [20–22].

Four OA receptors have been identified in *Drosophila*: Oamb, Octβ1R, Octβ2R, and Octβ3R. Oamb is most closely related to mammalian α-adrenergic receptors, and the other three to β-adrenergic receptors [17,23]. Recent work demonstrated that *Oamb* and *Octβ2R* are important in egg laying and ovulation [24–26]. Oamb is widely expressed in the female reproductive system, including the ovary, with strongest expression observed in the oviduct [24]. It is currently believed that OA activates receptors in the oviduct, inducing oviduct contraction and secretion, which ultimately regulates ovulation through an unknown mechanism [19,27,25]. In addition to OA signaling, ovulation in *Drosophila* is affected by female reproductive gland secretions [28] and by mating, which increases the ovulation rate by stimulating afferent nerve activity in the female reproductive tract [29–33,4]. In particular, Ovulin transferred into the female reproductive tract after mating was recently shown to increase octopaminergic signaling and relax oviduct muscle [34], consistent with the role of OA signaling in regulating muscle contraction. It is, however, not clear whether OA plays any direct roles in the ovary to control ovulation.

In addition to above important work on *Drosophila* ovulation (also see review [35]), recent studies from our lab also showed significant conservation of the basic cellular and molecular mechanisms of ovulation from flies to mammals. *Drosophila* female contains two ovaries that are connected by the oviduct. Each ovary is organized into ovarioles, which have mature follicles (stage-14 egg chambers) at the posterior end toward the oviduct (Fig 1A; [36]). Each mature follicle has one layer of epithelial follicle cells surrounding the oocyte. During ovulation, posterior follicle cells are first trimmed to break the follicle-cell layer and to allow the oocyte to be released into the oviduct. The rest of the follicle cells remain at the end of the ovariole and form a corpus luteum [37]. Similar to vertebrate ovulation [38–40], the entire follicle rupture requires matrix metalloproteinase 2 (Mmp2), a proteolytic enzyme expressed in posterior follicle cells of mature egg chambers but only activated during follicle rupture [37]. It is not yet clear what signals control Mmp2 activity, but it is clear that studying this question in *Drosophila* could yield important insights into the fundamental mechanism of ovulation.

Here, we developed the first *ex vivo* assay for follicle rupture in *Drosophila* and used it to investigate the role of octopaminergic signaling in this process. We found that OA directly activates its receptor Oamb on mature follicle cells to induce the breakdown of posterior follicle wall and ovulation. In addition, NE could partially substitute for OA, indicating an evolutionary conserved role for follicular adrenergic signaling in ovulation. Finally, we demonstrated that follicular adrenergic signaling activates Mmp2 activity to control ovulation via the intracellular Ca^2+^ as the second messenger. This is the first demonstration of a direct role of a neuromodulator in the control of follicle rupture during ovulation.

## Results

### Octopamine is sufficient to induce follicle rupture *ex vivo*

Octopaminergic neurons innervate ovarioles extensively [21], and OA receptor Oamb is transcribed in mature follicle cells according to *in situ* hybridization [24], microarray analysis (S1 Fig; [41]), and the expression of *R47A04-Gal4* [42], an *Oamb* enhancer element-regulated Gal4 driver, in mature follicle cells [37]. We examined whether OA activates Oamb directly in mature follicle cells to induce follicle rupture. Mature follicles with an intact layer of follicle cells marked by *R47A04-Gal4* were isolated from ovaries (see methods) and cultured with OA or control media (Fig 1A). After three hours, follicles in control medium maintained an intact follicle-cell layer (Fig 1B). In contrast, about 80% of the follicles cultured with 5 μM of OA had shed their follicle-cell layer to the dorsal appendage at the anterior tip of the oocytes (Fig 1C); some were completely detached from the oocyte and floating in the medium. This phenomenon of shedding the follicle-cell layer, which we called follicle rupture in our *ex vivo* culture system, is reminiscent of what occurs during the ovulation process *in vivo* [37]. The percentage of ruptured follicles with OA stimulation increased dramatically in the first two hours and reached a plateau at about three hours (Fig 1D). Extending the culture period neither increased the percent of ruptured follicles to 100% in the OA medium, nor allowed follicles in the control medium to reach the same level of rupture as OA-stimulated follicles (Fig 1D).

To validate that the follicle rupture in our *ex vivo* assay mimics the *in vivo* process, we video-recorded the entire rupturing process (Fig 1E and S1 Movie). We found that posterior follicle cells were first trimmed, as we previously observed *in vivo* [37]. The remaining follicle-cell layer was then squeezed toward the anterior dorsal appendage (Fig 1E and S1 Movie). The entire rupturing process took 13.1 ± 5.0 minutes (S1 Table), resembling the estimated *in vivo* ovulation time of 11.2 ± 2.5 minutes (Table 1; [37]). Each mature follicle initiated the follicle rupture asynchronously, likely reflecting their asynchronous developmental stages; however, the kinetics of all ruptures was similar, with a very slow initial speed (Fig 1F). It took about 10 minutes to rupture through the posterior half of the oocyte, but only four minutes for the rest of the area (Fig 1E and 1F).

**Table 1:** The effect of follicular adrenergic signaling on egg laying, egg distribution in the reproductive tract, and egg laying time.

Genotype	Egg laying in 2 days^#^		Egg distribution in 6h		Egg laying time (min)		
	N	Eggs/ female/ day	N	Uterus with egg (%)	Total time	Ovulation time	Uterus time
*Oamb* ^*MI12417*^ */TM3*	20	59.3 ± 2.8	76	55.3 ± 11.2	22.3 ± 1.1	10.0 ± 2.5	12.3 ± 2.6
*Oamb* ^*MI12417*^ */Df(3R)BSC141*	20	5.9 ± 3.6***	61	11.5 ± 8.0***	223.7 ± 136.5	198.0 ± 122.1	25.7 ± 23.8
*UAS-dcr2/+; 44E10-Gal4/+ (Ore-R)*	45	57.2 ± 2.7	118	40.7 ± 8.9	23.1 ± 1.1	13.7 ± 2.2	9.4 ± 2.1
*UAS-dcr2/+; 44E10-Gal4/+ (Attp2)*	50	72.4 ± 2.0*	118	44.9 ± 9.0	18.2 ± 0.5***	10.0 ± 1.7	8.2 ± 1.7
*UAS-dcr2/+; 44E10-Gal4/Oamb* ^*RNAi1*^	45	11.4 ± 1.2***	136	12.5 ± 5.6***	115.8 ± 12.1***	101.3 ± 12.4***	14.5 ± 6.6
*UAS-dcr2/+; 44E10-Gal4/Oamb* ^*RNAi2*^	25	21.3 ± 1.5***	76	26.3 ± 9.9*	62.1 ± 4.2***	45.8 ± 6.9***	16.3 ± 6.3
*UAS-dcr2/+; 44E10-Gal4/Oamb* ^*RNAi3*^	50	27.8 ± 2.2***	120	28.3 ± 8.1*	47.4 ± 3.7***	34.0 ± 4.7***	13.4 ± 4.0
*UAS-dcr2/+; 44E10-Gal4/Oamb* ^*RNAi4*^	25	31.0 ± 4.2**	26	23.1 ± 16.2	42.6 ± 5.7***	32.7 ± 8.2*	9.8 ± 7.0
*UAS-dcr2/+; 47A04-Gal4/+ (Ore-R)*	50	71.4 ± 1.6	51	39.2 ± 13.4	18.5 ± 0.4	11.2 ± 2.5	7.3 ± 2.5
*UAS-dcr2/+; 47A04-Gal4/Oamb* ^*RNAi1*^	45	29.3 ± 2.3***	67	16.4 ± 8.9**	45.1 ± 3.6***	37.7 ± 5.0***	7.4 ± 4.0
*UAS-dcr2/+; 47A04-Gal4/Oamb* ^*RNAi2*^	50	44.5 ± 1.5***	63	36.5 ± 11.9	29.6 ± 1.0***	18.8 ± 3.6	10.8 ± 3.5
*UAS-dcr2/+; 47A04-Gal4/Oamb* ^*RNAi3*^	50	53.5 ± 2.5**	65	21.5 ± 10.0*	24.7 ± 1.1***	19.4 ± 2.6*	5.3 ± 2.5
*UAS-dcr2/+; 47A04-Gal4/Oamb* ^*RNAi4*^	25	63.0 ± 3.4	32	34.4 ± 16.5	20.9 ± 1.1*	13.7 ± 3.5	7.2 ± 3.5

# one day = 22h at 29°C

* P<0.05

** P<0.01

*** P<0.001

All data are mean ± 95% confidence interval. Student's T-test was used for egg laying, Chi-square test was used for egg distribution, and Z Score test was used for egg laying time assuming normal distribution

To further examine the quality of *ex vivo* ruptured oocytes, we determined whether these oocytes were activated. Mature oocytes released into the oviduct are activated and resistant to bleach treatment because their egg shells are hardened through cross-linking [43]. This activation process can also be mimicked *in vitro* by culturing oocytes in hypotonic activation buffer [44,45]. Using the established bleach assay (see methods), we found that oocytes from our *ex vivo* assay dissolved immediately after bleach treatment (n = 96), indicating that they were not fully activated and their eggshells were not hardened. However, treatment with hypotonic activation buffer for 15 minutes can efficiently activate these ruptured oocytes (95%, n = 150; S2A and S2B Fig), indicating these oocytes from our *ex vivo* system are of good quality and responsive to egg activation stimuli.

OA-induced follicle rupture is dose-dependent. Stimulation with 1 μM of OA had a minimal effect on follicle rupture, while stimulation with 20 μM of OA reached the maximal effect (Fig 1G), which led us to use 20 μM for all the following experiments. In contrast, stimulation with 20 μM of tyramine (TA), the immediate precursor of OA, had a much weaker effect on follicle rupture (Fig 1H), consistent with a previous report that OA, but not TA, is responsible for inducing ovulation [20]. Since NE is the counterpart of OA in mammals, we tested whether NE can also induce follicle rupture in our *ex vivo* assay. NE had only a minimal effect at lower doses (Fig 1I). Higher doses of NE could induce follicle rupture (Fig 1I), likely reflecting a differential binding properties of OA and NE to their respective receptors [18]. Nevertheless, these data suggest that OA and NE play a conserved role in regulating follicle rupture. In summary, we developed the first *ex vivo* assay to study follicle rupture in *Drosophila*, and our data suggest that OA is sufficient to induce follicle rupture in the absence of the oviduct and muscle function, as these tissues were excluded from our culture assay (68 mature follicles examined and none had ovariole muscle; S3A and S3B Fig).

### Follicular Oamb is essential for OA/NE-induced follicle rupture

**Fig 2:**
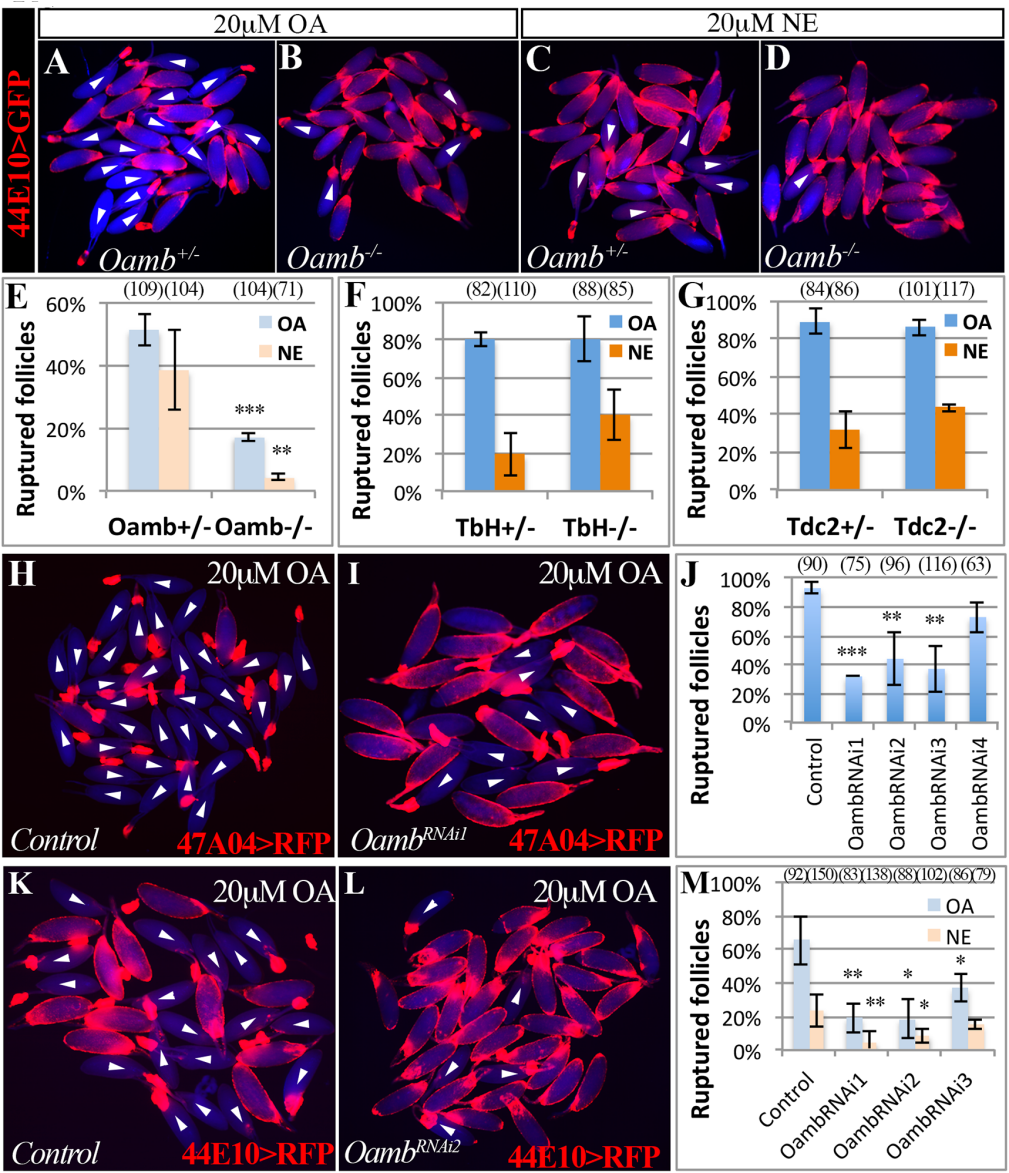
Follicular Oamb is required for OA/NE-induced follicle rupture. (A-D) Representative images show mature follicles (marked by *R44E10>GFP* in follicle cells in red) after three-hour culture with 20 μM of OA (A-B) or NE (C-D). Mature follicles are from control (A and C) and *Oamb* mutant (B and D) females. (E) Quantification of *Oamb* mutant mature follicles in response to OA or NE stimulation. Four replicates were used for each genotype, except in *Oamb* ^*-/-*^ group with NE treatment, which has three replicates. (F-G) Quantification of follicle rupture after three-hour OA or NE treatment (20 μM). Mature follicles were derived from *TβH* (F) or *Tdc2* (G) mutant females and marked by *47A04>RFP*. All treatments have three replicates except for *TβH* ^*+/-*^ with NE treatment and *Tdc2* ^*-/-*^, which have four replicates. (H-J) *Oamb* knockdown with *R47A04-Gal4* blocks follicle rupture. Representative images show control (H) and *Oamb* ^*RNAi1*^ (I) mature follicles after three-hour culture with 20 μM of OA. Quantification of follicle rupture (J). The number of replicates for each condition in (J) is 3, 3, 3, 4, and 2. (K-M) *Oamb* knockdown with *R44E10-Gal4* blocks follicle rupture induced by OA or NE. Representative images show control (K) and *Oamb* ^*RNAi2*^ (L) mature follicles after a three-hour culture with 20 μM of OA. Quantification of follicle rupture (M). The number of replicates for each condition in (M) is 6, 5, 4, and 3. Student’s T-test was used (*** P<0.001; ** P<0.01; * P<0.05).

To identify the receptor responsible for OA/NE-induced follicle rupture, we focused on Oamb, which is essential for ovulation [24] and is the most highly expressed OA receptor in mature follicles (S1 Fig). We verified the requirement of *Oamb* in ovulation with a new mutant allele (*Oamb* ^*MI12417*^), in which a MiMIC vector with a splice acceptor [46] was inserted in the coding intron of *Oamb* gene to disrupt the correct mRNA splicing (S4 Fig). Females bearing this mutant allele laid significantly fewer eggs and took a much longer time to ovulate an egg (Table 1). We then isolated mature follicles from these females and applied OA stimulation *ex vivo*. *Oamb* mutant follicles showed severe defects in OA-induced follicle rupture compared to control follicles (Fig 2A, 2B and 2E). In addition, the *Oamb* mutation abolished the NE-induced follicle rupture (Fig 2C–2E). The defective response of *Oamb* mutant follicles to OA/NE stimulation is not likely due to defective OA signaling in the oviduct or other organs, because follicles from *TβH* or *Tdc2* mutant females are fully competent to OA/NE-induced follicle rupture (Fig 2F and 2G). These data indicate that *Oamb* in mature follicles is likely responsible for OA/NE-induced follicle rupture.

To test if *Oamb* functions directly in mature follicle cells, we knocked down *Oamb* specifically in these cells with RNA interference (RNAi) and then performed OA stimulation *ex vivo*. *Oamb* knockdown in mature follicle cells with *R47A04-Gal4* severely disrupted OA-induced follicle rupture (Fig 2H–2J). Since *R47A04-Gal4* is regulated by an *Oamb* enhancer element [42], it could potentially be expressed in other *Oamb*-expressing cells, which may facilitate follicle maturation and ovulation. To exclude this possibility, we identified another Gal4 driver (*R44E10-Gal4*) expressed in mature follicle cells (S5B–S5D Fig). Compared to *R47A04-Gal4, which is only expressed in late stage-14 follicles (S5A Fig), R44E10-Gal4* was expressed in all stage-14 follicles, slightly earlier than *R47A04-Gal4*. *R44E10-Gal4* was not expressed in any tissues in the lower reproductive tract, nor in the neurons innervating the reproductive tract (S5B, S5E and S5F Fig). Like mature follicles isolated using *R47A04-Gal4*, follicles isolated using *R44E10-Gal4* were also responsive to OA/NE-induced follicle rupture (S5G and S5H Fig). In addition, mature follicles with *R44E10-Gal4* driving *Oamb* ^*RNAi*^ showed similar unresponsiveness to OA or NE stimulation (Fig 2K–2M). Taken together, these data suggest that follicular *Oamb* is required for OA/NE-induced follicle rupture *ex vivo*.

### Follicular adrenergic signaling is required for ovulation *in vivo*

**Fig 3:**
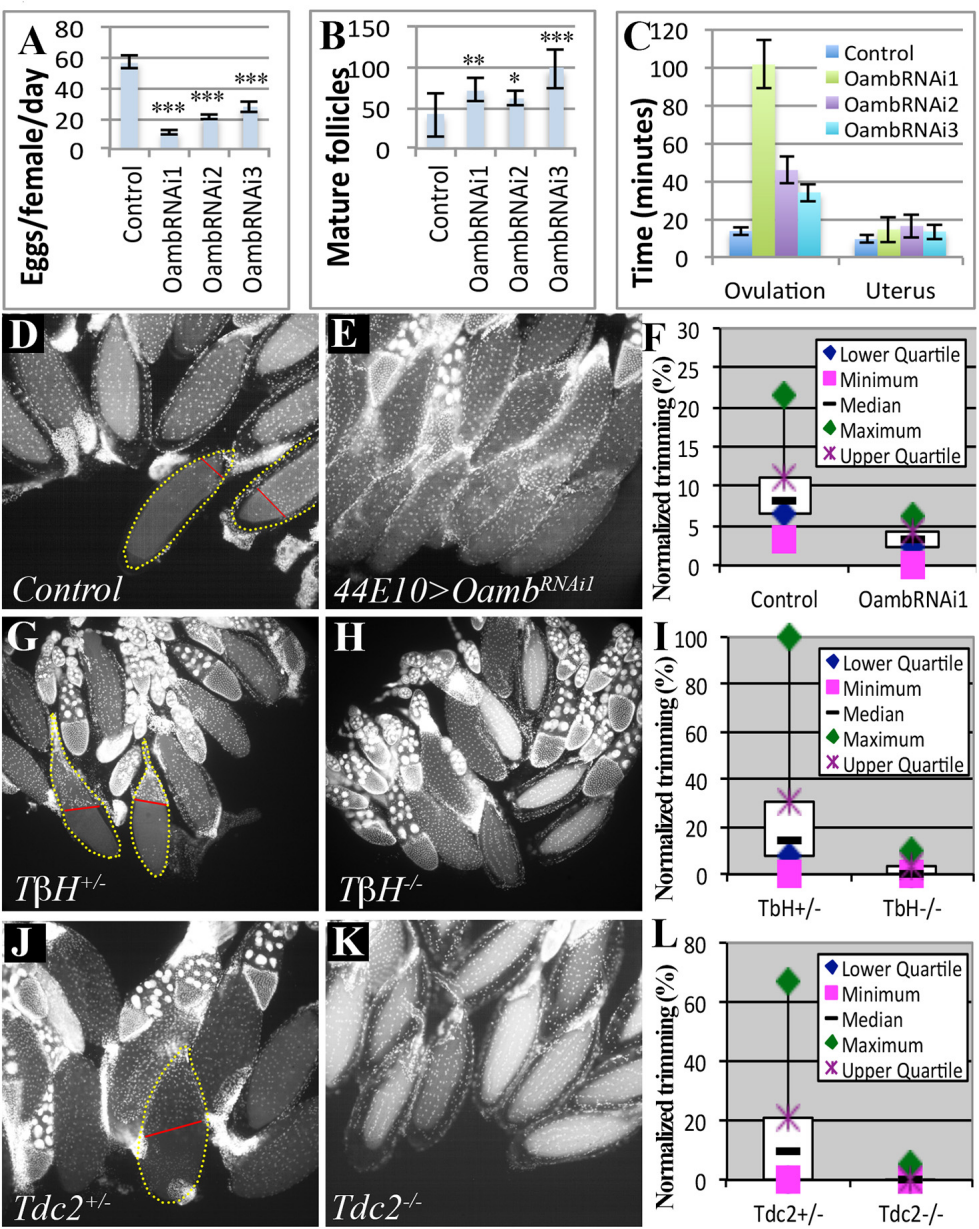
Follicular adrenergic signaling is required for ovulation and follicle cell trimming *in vivo*. (A-C) Egg laying (A), mature follicles in ovary (B), and the average ovulation and uterus time (C) is shown for control females or those expressing *Oamb* ^*RNAi*^ in mature follicle cells driven by *R44E10-Gal4*. Student’s T-test was used (A-B; *** P<0.001; **P<0.01; * P<0.05). (D-F) Follicle cell trimming is significantly reduced when follicular *Oamb* is knocked down by *R44E10-Gal4* driving *Oamb* ^*RNAi1*^ expression (*44E10>Oamb* ^*RNAi1*^). Representative images show trimmed follicles in control (D) but not *Oamb*-knockdown (E) ovaries. Trimmed follicles are outlined with dashed yellow lines, and the posterior leading edge of the follicle-cell layer is marked by a straight red line. Quantification of trimmed follicles (F). (G-L) Follicle cell trimming is also significantly reduced in *TβH* (G-I) or *Tdc2* (J-L) mutant females. See Tables 1 and 2 for the number of females analyzed and statistics.

To determine whether follicular adrenergic signaling is required for ovulation *in vivo*, we first analyzed the fecundity of females lacking follicular *Oamb*. Follicular *Oamb*-knockdown females with either *R47A04-Gal4* or *R44E10-Gal4* drivers laid significantly fewer eggs than control flies (Fig 3A and Table 1). The egg-laying defect is not caused by oogenesis problems, as mature follicles are abundant in these ovaries. In fact, *Oamb*-knockdown flies generally had more mature follicles in their ovaries (Fig 3B), indicating an ovulation defect. Indeed, *Oamb*-knockdown flies had a much longer ovulation time compared to control flies but did not show defects in transporting ovulated eggs into the uterus or ejecting them out of the uterus (Fig 3C and Table 1). These data strongly suggest that follicular *Oamb* is required for ovulation *in vivo*.

**Table 2:** The effect of follicular adrenergic signaling for follicle trimming.

Genotype	Mated or Virgin	No. of females	Mature eggs / female	Posterior trimmed eggs / female	Normalized trimming eggs / female (%)
*UAS-dcr2/+; 44E10-Gal4/+(Ore-R)*	6 hr mating	30	40.3 ± 21.5	3.7 ± 1.9	9.0 ± 3.9
*UAS-dcr2/+; 44E10-Gal4/Oamb* ^*RNAi1*^	6 hr mating	29	43.1 ± 12.1	1.4 ± 0.9 ***	3.1 ± 1.9 ***
*TDC* ^*RO54*^ */ Cyo*	6 hr mating	56	10.4 ± 11.6	1.3 ± 1.2	15.0 ± 16.5
*TDC* ^*RO54*^ */Df(2R)42*, *cn1*	6 hr mating	47	35.5 ± 11.6 ^#^	0.2 ± 0.4 ***	0.4 ± 1.1 ***
*TbH* ^*M18*^ */ FM7*	6 hr mating	20	20.1 ± 13.2	2.6 ± 1.3	21.4 ± 22.9
*TbH* ^*M18*^	6 hr mating	20	29.0 ± 8.4*	0.8 ± 1.2 ***	2.4 ± 3.6 ***
*TbH* ^*M18*^ */ FM7*	Virgin	20	35.3 ± 6.4	6.5 ± 2.7	18.6 ± 7.6
*TbH* ^*M18*^	Virgin	20	43.8 ± 11.5 **	1.0 ± 1.2 ***	2.0 ± 2.8 ***

* P<0.05

**P<0.01

***P<0.001

All data are mean ± SD. Student’s T-test was used.

Trimming of posterior follicle cells is essential for ovulation and precedes follicle rupture [37]. We investigated the role of follicular adrenergic signaling in this trimming process. Posterior trimmed follicles were readily observed in the ovaries of control females six hours after mating, and they account for 9% of the total mature follicles in each female (Fig 3D and 3F and Table 2), consistent with our previous analysis [37]. In contrast, the percentage of posterior trimmed follicles was reduced three fold in females lacking follicular *Oamb* (Fig 3E and 3F and Table 2), indicating its essential role in follicle trimming. This is consistent with our observation that posterior follicle cells remain intact in *Oamb*-knockdown follicles even after three hours of OA stimulation *ex vivo* (Fig 2I and 2L). Furthermore, the percentage of trimmed follicles also decreased in flies that lacked the ability to produce OA; we saw a reduction to 2.4% and 0.4% in *TβH* and *Tdc2* mutant females, respectively (Fig 3G–3L and Table 2). This reduction of trimmed follicles was not only observed in mated females, but also in virgin females (Table 2). Taken together, these data suggest that follicular adrenergic signaling is required for posterior follicle cell trimming.

### Adrenergic signaling activates Mmp2 to regulate ovulation

**Fig 4:**
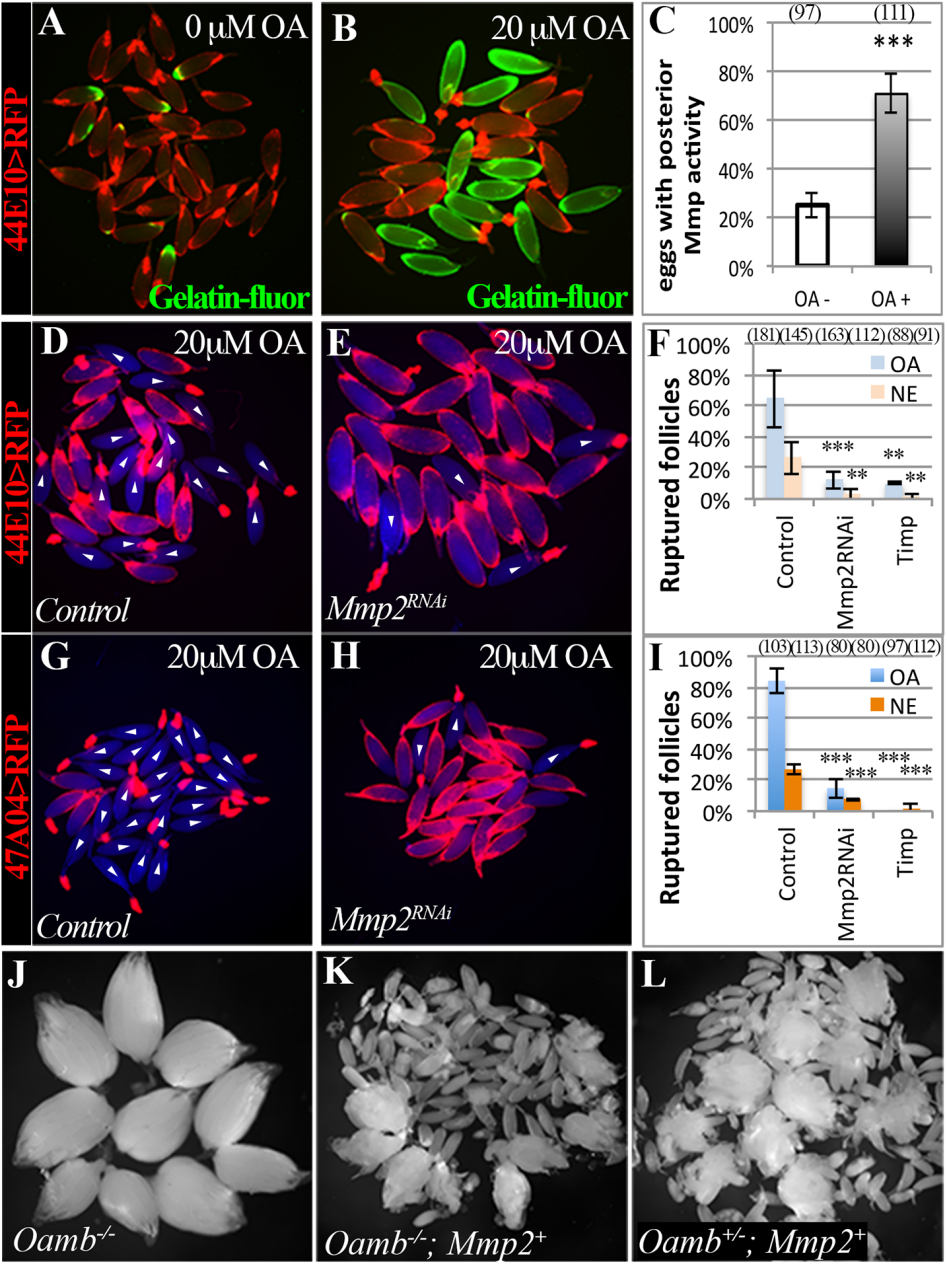
Adrenergic signaling activates Mmp2 to regulate ovulation. (A-C) *In situ* zymography shows increased Mmp activity in mature follicles after three-hour culture with 20 μM of OA. Mmp activity is indicated by Gelatin-fluorescein (green in A and B). The percentage of follicles with posterior Mmp activity is quantified in (C; *** P < 0.001). Three and four replicates were used for OA- and OA+ groups, respectively. (D-F) Expression of *Mmp2* ^*RNAi*^ or *Timp* driven by *R44E10-Gal4* prevents follicle rupture in response to OA or NE (*** P <0.001 and ** P < 0.01). The number of replicates used for each condition is 6, 5, 6, 4, 3, and 3. (G-I) Expression of *Mmp2* ^*RNAi*^ or *Timp* driven by *R47A04-Gal4* prevents follicle rupture in response to OA or NE. All experiments were performed in four replicates except *Mmp2* ^*RNAi*^, which have three replicates. (J-L) Ovaries are shown for the *Oamb* mutant (J), the *Oamb* mutant with ectopic expression of Mmp2 driven by *R44E10-Gal4* (K), and the *Oamb* heterozygous with ectopic Mmp2 expression (L). Mature eggs were released into the female abdominal cavity.

The crucial role of Mmp2 in trimming of posterior follicle cells [37] prompted us to investigate the relationship between follicular adrenergic signaling and Mmp2 activity. It is unlikely that adrenergic signaling regulates Mmp2 expression, as Mmp2 was readily detected in the posterior follicle cells of *TβH* mutants (S6A and S6B Fig). To test whether OA regulates Mmp2 activity, we examined gelatinase enzymatic activity in the OA-induced *ex vivo* ovulation assay using *in situ* zymography [37,38]. About 20% of mature follicles cultured in a control medium had gelatinase activity at their posterior end (Figs 4A, 4C, S6C and S6G). In contrast, more than 70% of mature follicles stimulated with OA had gelatinase activity (Figs 4B, 4C, S6D and S6G). The entire eggshells of ruptured oocytes were coated with Mmp-activated gelatin-fluorescein (Figs 4B and S6D), as we observed *in vivo* [37]. In addition, OA-induced gelatinase activity was blocked in mature follicles with *Oamb* knockdown or misexpression of *Timp*, an endogenous inhibitor of Mmp2 [47], in follicle cells (S6E–S6G Fig). These data indicate that OA-Oamb signaling is sufficient to induce Mmp2 activation.

To determine whether Mmp2 activity is required for OA-induced follicle rupture, we isolated mature follicles containing follicle cell-specific Mmp2 knockdown and cultured them in the OA medium. These follicles did not respond to OA stimulation, and their posterior follicle cells remained intact (Fig 4D–4I). In addition, Mmp2 knockdown in follicle cells also abolished the NE-induced follicle rupture (Fig 4F and 4I). Furthermore, misexpression of Timp in mature follicle cells completely prevented follicle rupture *ex vivo* (Fig 4F and 4I). Therefore, Mmp2 activity in mature follicle cells is essential for OA/NE-induced follicle rupture *ex vivo*, consistent with its essential role in follicle trimming and ovulation *in vivo* [37].

To confirm that Mmp2 acts downstream of adrenergic signaling in follicle trimming and rupture, we attempted to rescue the defect of follicle rupture in *Oamb* mutant flies with ectopic expression of Mmp2 in mature follicle cells. *Oamb* mutant females had two intact ovaries, which contain a large number of mature follicles (Fig 4J). In contrast, follicular misexpression of Mmp2 in *Oamb* mutant females caused the breakdown of the ovariole muscle sheath and the release of mature follicles into the abdominal cavity (Fig 4K). Further examination of these released follicles demonstrated that 99% of them (n = 70) had no follicle-cell covering, similar to follicles released upon misexpression of Mmp2 in *Oamb* heterozygous or wild-type females (Fig 4L; [37]). Therefore, Mmp2 is sufficient to induce follicle rupture in the absence of adrenergic signaling. Together, our data indicate that follicular adrenergic signaling activates Mmp2 to control follicle trimming and ovulation.

### Intracellular Ca^2+^ acts as the second messenger downstream of follicular adrenergic signaling to induce follicle rupture

**Fig 5:**
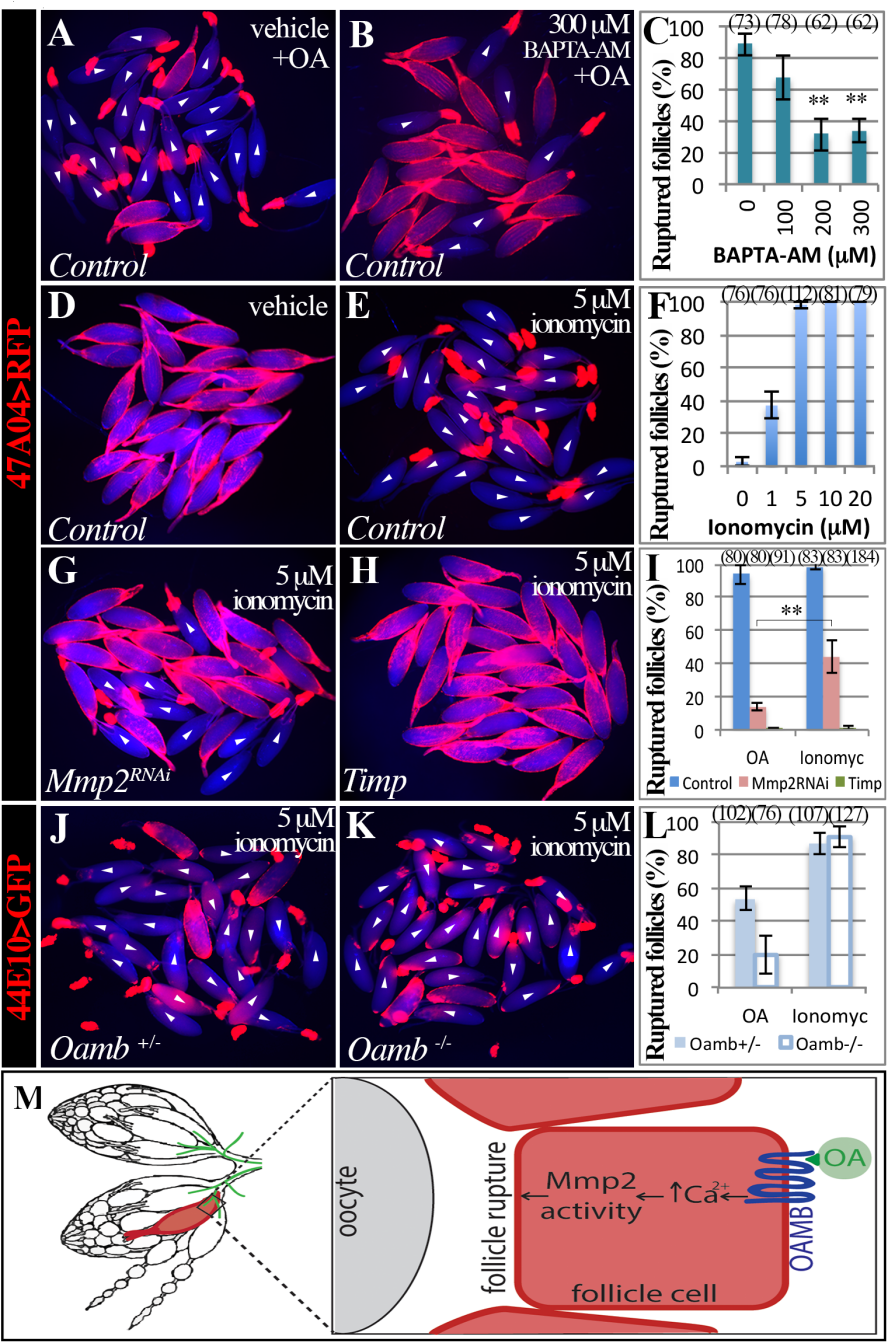
Intracellular Ca^2+^ is the second messenger downstream of follicular adrenergic signaling. (A-C) Pretreatment of BAPTA-AM blocks OA-induced follicle rupture. Representative images show mature follicles treated with DMSO (A) or BAPTA-AM (B) followed a three-hour stimulation with 20 μM of OA. Ruptured follicles were quantified in C. Three replicates are used for each condition. (D-F) Ionomycin is sufficient to induced follicle rupture. Representative images show follicles after three-hour culture with ethanol (D) or 5 μM of ionomycin. Ruptured follicles after different doses of ionomycin treatment are quantified in F. All conditions have three replicates except in 5 μM, which has four replicates. (G-H) Representative images of *Mmp2*-knockdown (G) and *Timp*-overexpressing (H) follicles treated with 5 μM of ionomycin for three hours. (I) Quantification of ruptured follicles with Mmp2 knockdown or Timp overexpression in mature follicle cells in response to 20 μM of OA or 5 μM of ionomycin stimulation. All conditions have three replicates except for Timp overexpression with ionomycin treatment, which has six replicates. (J-L) Ionomycin, but not OA, is sufficient to induce rupture in *Oamb* mutant follicles. Representative images show *Oamb* ^*+/-*^ (J) and *Oamb* ^*-/-*^ (K) follicles after three-hour culture with ionomycin. (L) Quantification of ruptured follicles after three-hour culture with 20 μM of OA or 5 μM of ionomycin. The number of replicates for each condition is 4, 4, 3, and 5. (M) A cartoon showing the model of follicular adrenergic signaling in Mmp activity and follicle rupture. Octopaminergic neurons are shown in green.

OA-Oamb interaction can induce transient increase of intracellular Ca^2+^ concentration ([Ca^2+^]_i_) [23]. To determine whether OA evokes Ca^2+^ signaling in mature follicle cells to induce follicle rupture, we first monitored the [Ca^2+^]_i_ using a genetically encoded calcium sensor (see methods). Fluorescent intensity of the calcium sensor expressed in mature follicle cells rose significantly around six minutes after OA administration in our *ex vivo* culture system (S7 Fig and S2 Movie). To determine whether Ca^2+^ is required for OA-induced follicle rupture, we pretreated mature follicles with BAPTA-AM, an intracellular Ca^2+^ chelator, before OA stimulation. Two hundred μM BAPTA-AM treatment significantly perturbed the OA-induced follicle rupture (Fig 5A–5C). To determine whether Ca^2+^ is sufficient to induce follicle rupture, we stimulated mature follicles with ionomycin, a potent ionophore for increasing [Ca^2+^]_i_. Ionomycin is potent to induce follicle rupture even at 5 μM concentration (Fig 5D–5F), lower than the dose typically used in the field [48]. Taken together these data suggest that the increase of [Ca^2+^]_i_ is both necessary and sufficient to induce follicle rupture.

To further test whether Ca^2+^ is the second messenger of follicular adrenergic signaling for Mmp2 activation and follicle rupture, we set to examine whether ionomycin is sufficient to induce rupture of follicles lacking follicular *Mmp2* or *Oamb*, which do not respond to OA stimulation. Ionomycin only partially induces follicle rupture when Mmp2 is knocked down in mature follicle cells and is not able to induce any rupture when Timp is overexpressed (Fig 5G–5I). In contrast, ionomycin is able to induce follicle rupture in both control and *Oamb* mutant follicles at the equal efficiency (Fig 5J–5L). All these data indicate that Ca^2+^ acts downstream of Oamb but upstream of Mmp2 during follicle rupture. Together, we conclude that follicular adrenergic signaling activates Mmp2 to control follicle trimming and ovulation likely via intracellular Ca^2+^ (Fig 5M).

## Discussion

### The first *ex vivo* follicle rupture assay in *Drosophila*

Ovulation, an essential step in metazoan reproduction, has been extensively studied in mammals over the past several decades [49–51]. However, progress in the field has been hindered by the limited ability of mammalian model systems to be genetically manipulated. Thus it is still unclear how follicles break their wall in a highly regulated spatio-temporal manner to allow release of oocytes. The model organism *Drosophila* offers a wealth of tools for genetic manipulation, but to date, few specific readouts for *Drosophila* ovulation has been developed. Previous studies of *Drosophila* ovulation have used readouts such as egg laying, percentage of females with eggs in the reproductive tract, or egg retention [21,25–27,33]. We recently combined these parameters to estimate ovulation time [28,37]. In the present study, we developed the first *ex vivo* follicle rupture assay in *Drosophila* and demonstrated that OA-induced follicle rupture in this assay is similar to the rupturing process *in vivo*. This assay gave us the unprecedented ability to visualize the entire process of follicle rupture and quantify its kinetics. Further genetic evidence illustrated that genes required for *ex vivo* follicle rupture are also required for *in vivo* ovulation, including *Oamb* and *Mmp2*. Our *ex vivo* assay represents a simple, specific, and reliable method for measuring rupturing ability of mature follicles. In conjunction with the powerful genetic tools available in *Drosophila*, this *ex vivo* assay will allow genetic screens to identify candidate genes involved in follicle rupture, thus opening new avenues for ovulation research.

### A direct role for octopamine signaling in *Drosophila* ovulation

Octopamine, a biogenic amine derived from tyrosine, has been identified as essential for ovulation in *Drosophila* [20]. The major source of OA is octopaminergic neurons innervating the female reproductive system, and previous studies showed that restoring *TβH* specifically in these neurons rescues the ovulation defect caused by *TβH* mutation [21]. Due to its effects on muscle contraction, OA was proposed to regulate ovulation by inducing the contraction of ovarian muscle and relaxation of oviduct muscle [3,19,25,27,34].

Ovarian smooth muscle contraction was also proposed to regulate ovulation in mammals in the early 1980’s [11,52,53]. However, subsequent work suggest that ovulation requires the active proteolytic degradation of the follicle wall rather than passive muscle contraction [54,40,55]. At least three families of proteolytic enzymes are involved in this process, including matrix metalloproteinases [56,57]. Pharmacological blockage of any of these enzymes results in inhibition of follicle rupture.

Our recent work suggested that *Drosophila* also requires proteolysis for breaking the follicle wall and ovulation [37], and in this way shares similarities with mammalian ovulation at both the cellular and molecular level [28,37]. These new insights into *Drosophila* ovulation process lead to the speculation that octopaminergic signaling may play a direct role on the follicle in controlling ovulation in addition to its role on muscle contraction. Here, we demonstrate that OA-Oamb signaling in mature follicle cells directly regulates follicle wall degradation, follicle rupture, and ovulation by activating key enzyme Mmp2. Furthermore, our pharmacological data suggest that OA-Oamb signaling likely fulfill these functions via intracellular Ca^2+^ as the second messenger. However, it is still unclear how OA-Oamb-Ca^2+^ regulates Mmp2 activity. Lacking a method to detect Mmp2 protein prevents us to test whether OA-Oamb-Ca^2+^ regulates Mmp2 protein secretion. The *Mmp2*::*GFP* fusion allele we previously generated [37] is good to detect Mmp2::GFP expression but not good to track Mmp2 secretion because Mmp2::GFP fusion proteins are not properly processed and secreted to the extracellular space (S8 Fig) and *Mmp2*::*GFP* homozygous flies are lethal as Mmp2 mutant females do [47]. Alternatively, Ca^2+^ signaling may indirectly regulate Mmp2 activity via its inhibitor or other regulatory processes. Despite that, it is intriguing that [Ca^2+^]_i_ also rises after NE and gonadotropin stimulation in human granulosa cells [58] and that perfusion of a Ca^2+^ chelator in rabbits significantly reduces gonadotropin-induced ovulatory efficiency [59]. Given adrenergic innervation of ovaries observed throughout metazoans, it is plausible to speculate that follicular adrenergic signaling plays conserved roles in regulating Mmp activity and ovulation (See below).

### Conservation of ovarian adrenergic signaling in ovulation

Adrenergic innervation of the ovary has long been found in mammals including humans. The role of adrenergic signaling in ovulation has been studied as early as the 1970’s. The neurotransmitter norepinephrine (NE) reaches the highest level in peripheral plasma during ovulation [60] and is enriched in the follicular fluid of preovulatory follicles compared to in peripheral plasma in healthy women [16,61,62]. Functional adrenergic receptors are expressed in mammalian ovarian follicular cells [13,58,63]. Ovarian perfusion of adrenergic agonists or antagonists influences the ovulation rate in rabbits and rats [12,14]. It has been speculated that adrenergic signaling regulates ovulation by stimulating muscle contraction or by increasing production of reactive oxygen species [16,53]. In contrast to this view, ovarian sympathetic denervation does not affect ovulation in rabbits and rats [10,15]; instead, it rescues ovulation defect in a rat model of PCOS [64,65], which is associated with increased sympathetic inputs to the ovary [8,9]. It is not clear why a discrepancy exists between the effects of surgical denervation and of pharmacological agents. Thus, no consensus has been reached in regard to the role of ovarian adrenergic signaling in mammalian ovulation.

Instead of regulating ovarian smooth muscle contraction, the results of the present study suggest an alternative pathway for ovarian NE to regulate ovulation. NE likely activates adrenergic receptors in granulosa and theca cells (equivalent to *Drosophila* follicle cells) in mammalian periovulatory follicles, which activates Mmp enzymatic activity at the apex [38], where mature oocytes rupture through. A surgical denervation may cause tissue damage and activate Mmps directly, bypassing the requirement of follicular adrenergic signaling. Future studies, using both mammalian and *Drosophila* genetic tools, will identify fundamental mechanisms of adrenergic signaling in ovulation.

## Materials and Methods

### *Drosophila* genetics

Flies were reared on standard cornmeal-molasses food at 25°C unless otherwise indicated. *Oamb* ^*MI12417*^ is a MiMIC line inserted in the coding intron of both *Oamb* spicing isoforms (S4 Fig) [46], and *Oamb* ^*MI12417*^ */Df(3R) BSC141* was used to characterize the *Oamb* mutant phenotype. *TbH* ^*M18*^ [20] and *Tdc2* ^*RO54*^ [22] were kindly provided by Dr. Mariana Wolfner. All RNAi-knockdown experiments were performed at 29°C with *UAS-dcr2* to increase the efficiency of RNAi. *R47A04-Gal4* (*Oamb*) and *R44E10-Gal4* (*lilli*) from the Janelia Gal4 collection [42] were used for misexpressing genes or RNAi in mature follicle cells. The following RNAi or overexpressing lines were used: *UAS-Oamb* ^*RNAi1*^ (V2861) and *UAS-Oamb* ^*RNAi2*^ (V106511) from the Vienna *Drosophila* Resource Center; *UAS-Oamb* ^*RNAi3*^ (B31233) and *UAS-Oamb* ^*RNAi4*^ (B31171) from the Bloomington *Drosophila* Stock Center; UAS-Mmp2^RNAi^ [66]; UAS-Mmp2 [47]; and *UAS-GCaMP5G* [67]. *UASpGFP-act79B; UAS-mCD8-GFP*[37] was used to analyze Gal4 expression in both germline and somatic cells, as well as neurons. *UAS-GFPnls* and *UAS-RFP* were used for follicle isolation. Control flies were derived from specific Gal4 drivers crossed to Oregon-R or *yv; attP2* (B36303). The *Mmp2*::*GFP* fusion allele in the *Mmp2* endogenous locus was used for detecting Mmp2 protein expression [37].

### *Ex vivo* follicle rupture, Ca^2+^ imaging, *in situ* zymography, and egg activation assays

For the *ex vivo* follicle rupture assay, 4–6-day-old virgin females were used to isolate mature follicles, and follicle cells were fluorescently labeled using *R47A04-Gal4* or *R44E10-Gal4*. Ovaries were dissected in Grace’s medium and ovarioles were separated from each other using forceps. This process will partially break the ovariole muscle sheath and release mature follicles. Mature follicles with an intact follicle-cell layer and completely dissociated from younger follicles were immediately transferred to new Grace’s medium to minimize their exposure to endogenous biogenic amines during dissection. With this method, we can isolate about 10 mature follicles/female and isolated mature follicles are no longer surrounded by ovariole or oviduct muscle sheaths (S3 Fig). Within one hour, isolated mature follicles were subsequently cultured in culture media (Grace’s medium, 10% fetal bovine serum, and 1X penicillin/streptomycin) supplemented with the indicated concentration of OA, TA, NE (Sigma), or ionomycin (dissolved in ethanol; Cayman Chemical). For chelating intracellular Ca^2+^, isolated mature follicles were treated with BAPTA-AM (dissolved in DMSO; Cayman Chemical) for 30 minutes before OA culture. All cultures were performed at 29°C, the same condition as flies were maintained, to enhance Gal4/UAS expression. About 25–30 follicles were used for each culture group and the percentage of ruptured follicles was then calculated as one data point. Typically three-six replicates were used for each genotype or treatment; data were represented as mean percentage ± standard deviation (SD); and Student’s T-test was used for statistic analysis. Ruptured follicles were defined as those losing more than 80% follicle-cell covering. With the exception of Fig 1D, all data were collected at the end of the three-hour culture.

For Ca^2+^ imaging and follicle rupture kinetics, video images were captured at 0.2 frame/second (FPS) with a sCOMS camera (PCO.Edge) installed in a Leica MZ10F fluorescent stereoscope. To examine the kinetics of follicle rupture, mature follicles were cultured in 20 μM of OA medium for 20 minutes at 29°C before recording. Unruptured follicles were then transferred into a home-made slide for video recording at room temperature. Each ruptured follicle was analyzed frame-by-frame manually to determine the ruptured distance between the posterior tip of the oocyte and the posterior leading edge of the follicle-cell layer using ImageJ. The percent of ruptured distance was then calculated as the ruptured distance divided by the length of the oocyte from the anterior to posterior tip. Because of the asynchronous onset of follicle rupture, data were normalized at the time point when follicles reach 50% ruptured distance.

*In situ* zymography for detecting gelatinase activity was performed as previously reported with minor modifications [37]. 50 μg/ml of DQ-gelatin conjugated with fluorescein (Invitrogen) was added into the culture media with or without OA for three hours. After a quick rinse, mature follicles with posterior fluorescent signal were directly counted. For egg activation, ruptured oocytes were treated with hypotonic activation buffer [45] for 15 minutes and treated with 50% bleach for three minutes. The number of unbroken oocytes was counted.

### Egg laying, ovulation time, and follicle cell trimming

Egg laying, ovulation time, and follicle cell trimming were performed as previously described [28,37]. In brief, 4–6-day-old virgin females fed with wet yeast for one day were used. For egg laying, five females were housed with ten Oregon-R males in one bottle to lay eggs on grape juice-agar plates for two days at 29°C. After egg laying, ovaries were dissected and mature follicles in these ovaries were counted. The number of eggs on the plates was then counted, which was used to calculate the average time for laying an egg (egg-laying time). The egg-laying time was partitioned into the ovulation time and the uterus time (the time egg spent in the uterus and during oviposition). The partition ratio was determined based on the percentage of females having eggs in the uterus at six hours after mating. To do so, ten virgins were placed in a vial with 15 *Oregon R* males for six hours at 29°C, frozen for 4.5 minutes at -80°C, and then dissected to examine the reproductive tract. For follicle cell trimming, virgin or mated females were frozen for 4.5 minutes at -80°C, and ovary pairs were dissected, fixed, stained with DAPI, and mounted carefully to preserve the posterior end of mature follicles. Trimmed follicles were defined as more than a quarter of oocytes at the posterior end lacking follicle cell covering. Normalized trimming follicles were then calculated by the number of trimming follicles divided by the number of mature follicles in each female.

### Immunostaining and microscopy

Immunostaining was performed following a standard procedure [68], including fixation in 4% EM-grade paraformaldehyde for 15 minutes, blocking in PBTG (PBS+ 0.2% Triton+ 0.5% BSA+ 2% normal goat serum), and primary and secondary antibody staining. For Mmp2::GFP localization, dissected tissues were stained in primary antibodies for 45 minutes at 4°C before the fixation treatment followed with the secondary antibody staining. Mouse anti-Hnt (1:75; Developmental Study Hybridoma Bank) and rabbit anti-GFP (1:4000; Invitrogen) were used as primary antibodies, and Alexa 488 goat anti-rabbit and 546 goat anti-mouse (1:1000, Invitrogen) were used as secondary antibodies. Images were acquired using a Leica TCS SP8 confocal microscope or Leica MZ10F fluorescent stereoscope with a sCOMS camera (PCO.Edge), and assembled using Photoshop software (Adobe, Inc.).

## Supporting Information

S1 Fig Expression of OA/TA receptors in stage 10, 12, and 14 follicles. Data were mined from previous microarray analysis [41]. Two independent datasets of stage 10 and 14 follicles were used for calculating mean expression and standard deviation.
(TIF)

S2 Fig Ruptured oocytes can be activated by hypotonic buffer. (A-B) Hypotonic buffer-treated ruptured follicles before (A) and after (B) bleach treatment. Eggs tolerant to bleach treatment were activated.
(TIF)

S3 Fig Isolated mature follicles do not contain ovariole muscle sheath. (A) Intact ovaries stained with phalloidin (green in A and white in A’) show ovariole muscle sheath wrapping around the ovarioles. (B) Isolated mature follicles stained with phalloidin (green in B and white in B’) are not surrounded by the ovariole muscle sheath.
(TIF)

S4 Fig Molecular characterization of *Oamb* ^*MI12417*^ allele. The upper panel shows the genomic organization and alterative splicing of *Oamb* gene. The MiMIC insertion in *Oamb* ^*MI12417*^ allele is also indicated. Orange boxes depict the coding exons and red arrows indicate PCR primer. The bottom panel shows RT-PCR results using isolated mature follicles. *Oamb* ^*K3*^, but not *Oamb* ^*AS*^, is expressed in *Oamb* ^*MI12417*^ */+* mature follicles (Lane 3 and 5), consistent with previous report[24]. In contrast, neither of these isoforms are expressed in *Oamb* ^*MI12417*^ */Df(3R) BSC141* mature follicles (Lane 4 and 6). The primers used were: CCGCTTCAAGGGACAGTATC (rp49-F), GACAATCTCCTTGCGCTTCT (rp49-R), TGACCAACGATCGGGGTTAT (K3-F), ATGCGCAATATGAGCTGGGA (K3-R), AGAACGACGAGAGCCATCAA (AS-F), TTGATCTTGTCGTGGTGGTG (AS-R).
(TIF)

S5 Fig Expression of *R47A04-Gal4* and *R44E10-Gal4* and *R44E10-Gal4*-labeled follicles in response to OA and NE. (A) *R47A04-Gal4* driving *UAS-GFP* expression (47A04>GFP) in follicle cells of late, but not early, stage-14 egg chambers. Early stage-14 egg chambers are recognized based on remnant of nurse-cell nuclei (arrows). (B-F) *R44E10-Gal4* expression (*44E10>GFP*) in the female reproductive system. *R44E10-Gal4* is expressed in follicle cells of all stage-14 egg chambers (B and D), but not in younger egg chambers (B and C). It is not expressed in any region of the oviduct (B, E and F), nor in the uterus, spermathecae, or neurons innervating the reproductive tract (B and F). The oviduct is outlined by a white line in E and F and an asterisk in B. The oocyte halfway in the oviduct is outlined by a dashed yellow line, and the posterior leading edge of the follicle-cell layer is marked by a red line in E. An arrow points to the spermathecae in F. Hnt (red) is an zinc-finger transcription factor expressed in mature follicle cells [37] and spermathecal glands [28]. (G-H) The dose response of *R44E10-Gal4*-labeled mature follicles to OA (G) and NE (H) in follicle rupture. The reduced response with *R44E10-Gal4* labeling than *R47A04-Gal4* is likely because it enables the isolation of slightly early stage-14 egg chambers. All conditions have three replicates except 0 and 20 μM OA, which have five replicates.
(TIF)

S6 Fig Follicular adrenergic signaling activates Mmp2 enzymatic activity but not Mmp2 expression. (A-B) Mmp2::GFP is expressed normally in posterior follicle cells of control (A) and *TβH* mutant (B) follicles. (C-F) Gelatinase activity in mature follicles after three-hour cultures without (C) or with (D-F) 20 μM of OA. Mature follicles were from control females (C-D) and females with *47A04-Gal4* driving *Oamb* ^*RNAi1*^ (E) and *Timp* (F) expression. (G) Quantification of gelatinase activity from (C-F). ** P<0.01. All conditions have three replicates.
(TIF)

S7 Fig Intracellular Ca^2+^ concentration increases after OA stimulation. Ca^2+^ flux detected by *R47A04-Gal4* driving *UAS-GCaMP5G* in mature follicle cells. The zero time point is 15 second before OA administration. The signal intensity was maximum around 6:30 (mm:ss). Fluorescence intensities are presented using a false-color scale, shown in the first panel.
(TIF)

S8 Fig Mmp2::GFP fusion protein is trapped intracellularly. (A-A’) GFP antibody is applied after the fixation to permeabilize the cell membrane. Mmp2::GFP is detected in posterior follicle cells. (B-C’) GFP antibody is applied before the fixation to label the extracellular Mmp2::GFP. Mmp2::GFP is not detected in posterior follicle cells without (B-B’) and with (C-C’) OA stimulation. Together with the fact that *Mmp2*::*GFP* homozygous females are lethal, this result indicate that Mmp2::GFP fusion proteins are trapped inside the cell.
(TIF)

S1 Movie Follicle rupture after 20 μM of OA stimulation. (AVI)

S2 Movie Ca^2+^ flux in mature follicle cells after 20 μM of OA stimulation. (AVI)

S1 Table The analysis of kinetics of *ex vivo* follicle rupture. (DOCX)
