# Supplementary figures and images for: A Follicle Rupture Assay Reveals an Essential Role for Follicular Adrenergic Signaling in Drosophila Ovulation
Source: PLoS Genet. 2015 Oct 16;11(10):e1005604. doi: 10.1371/journal.pgen.1005604 (PMC4608792; doi:10.1371/journal.pgen.1005604)

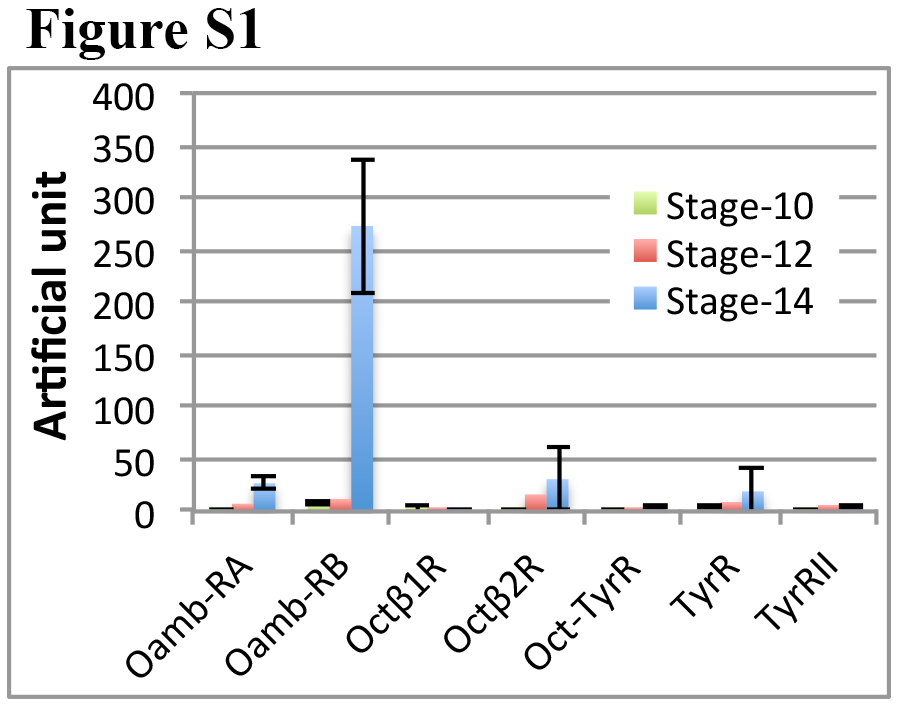

Supplement: S1 Fig — Data were mined from previous microarray analysis [41]. Two independent datasets of stage 10 and 14 follicles were used for calculating mean expression and standard deviation. (TIF) [file pgen.1005604.s001.tif]

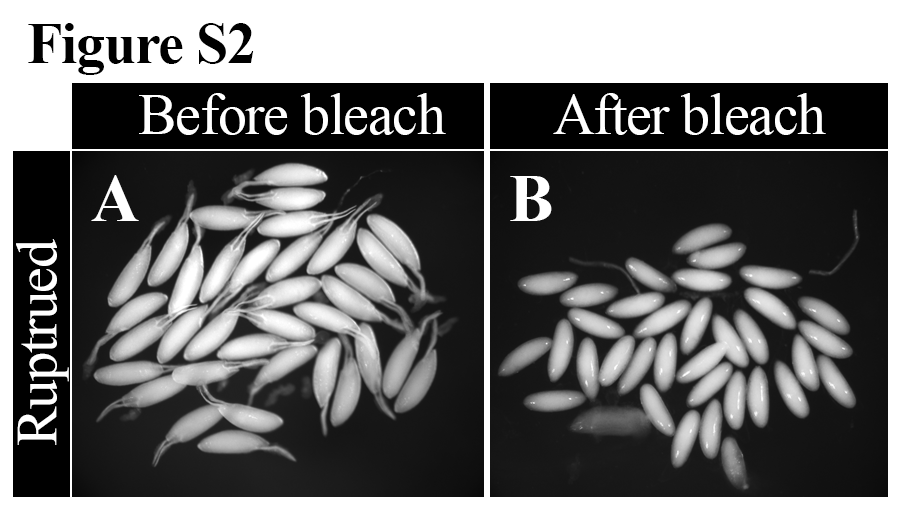

Supplement: S2 Fig — (A-B) Hypotonic buffer-treated ruptured follicles before (A) and after (B) bleach treatment. Eggs tolerant to bleach treatment were activated. (TIF) [file pgen.1005604.s002.tif]

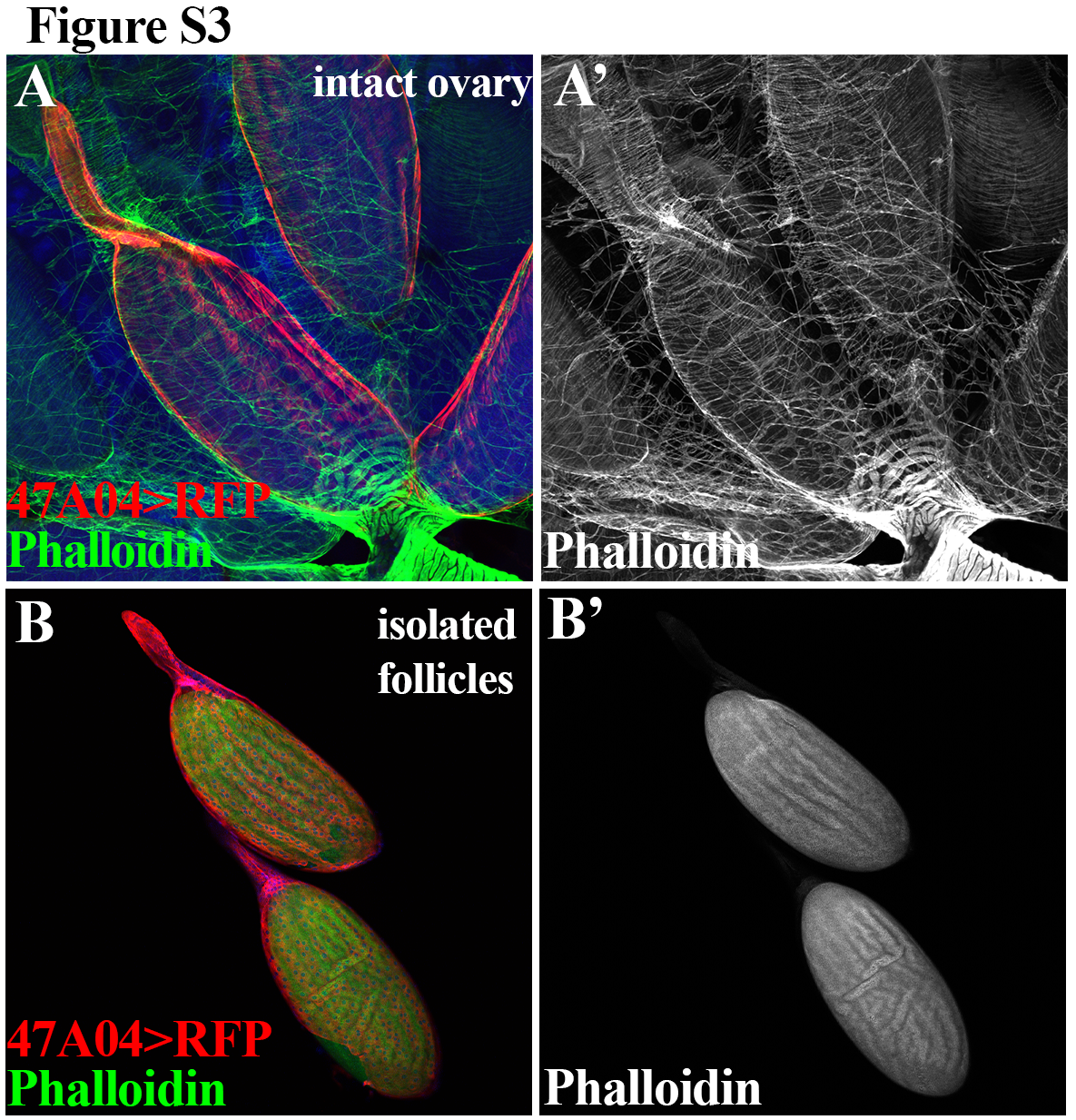

Supplement: S3 Fig — (A) Intact ovaries stained with phalloidin (green in A and white in A’) show ovariole muscle sheath wrapping around the ovarioles. (B) Isolated mature follicles stained with phalloidin (green in B and white in B’) are not surrounded by the ovariole muscle sheath. (TIF) [file pgen.1005604.s003.tif]

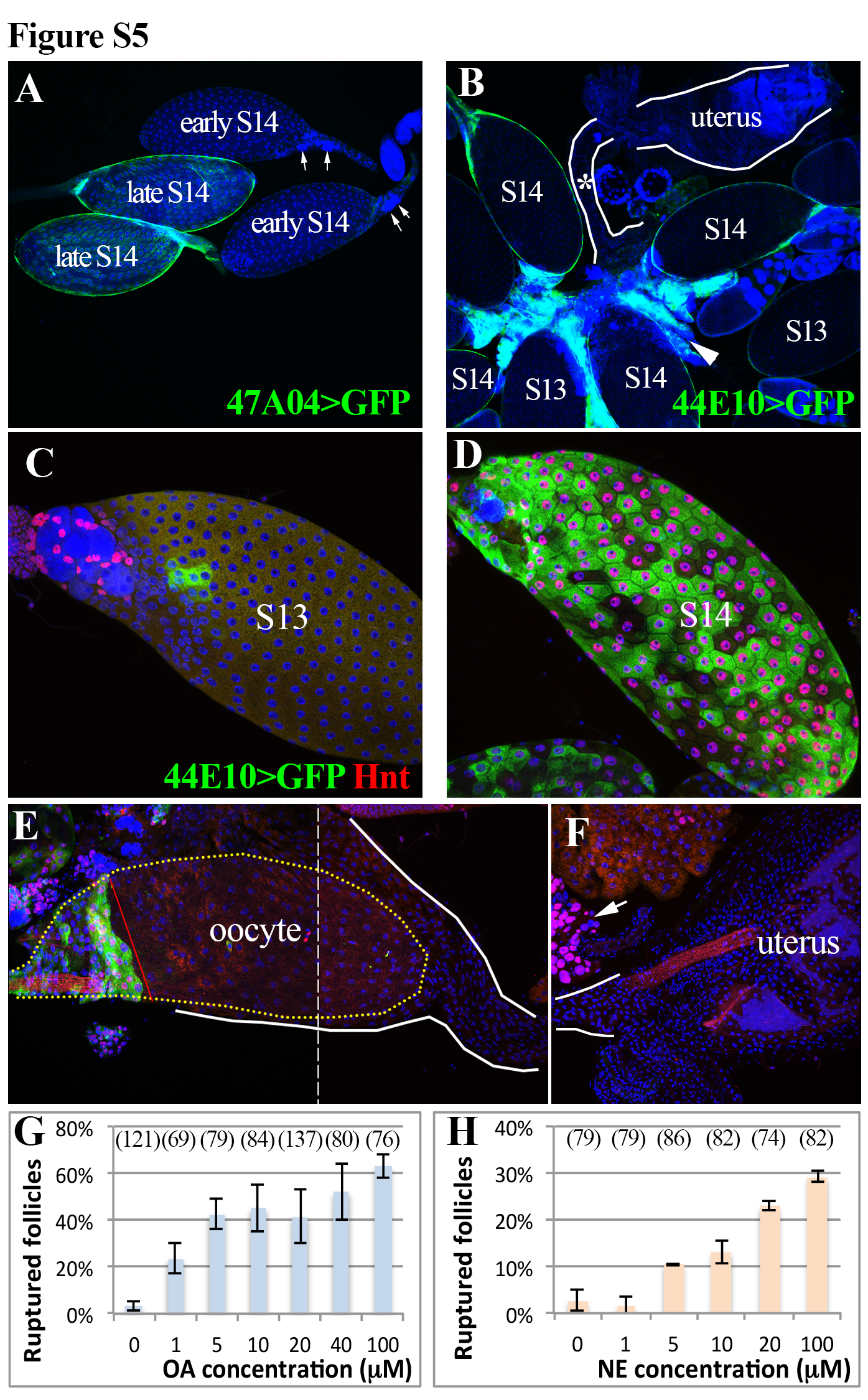

Supplement: S5 Fig — (A) R47A04-Gal4 driving UAS-GFP expression (47A04>GFP) in follicle cells of late, but not early, stage-14 egg chambers. Early stage-14 egg chambers are recognized based on remnant of nurse-cell nuclei (arrows). (B-F) R44E10-Gal4 expression (44E10>GFP) in the female reproductive system. R44E10-Gal4 is expressed in follicle cells of all stage-14 egg chambers (B and D), but not in younger egg chambers (B and C). It is not expressed in any region of the oviduct (B, E and F), nor in the uterus, spermathecae, or neurons innervating the reproductive tract (B and F). The oviduct is outlined by a white line in E and F and an asterisk in B. The oocyte halfway in the oviduct is outlined by a dashed yellow line, and the posterior leading edge of the follicle-cell layer is marked by a red line in E. An arrow points to the spermathecae in F. Hnt (red) is an zinc-finger transcription factor expressed in mature follicle cells [37] and spermathecal glands [28]. (G-H) The dose response of R44E10-Gal4-labeled mature follicles to OA (G) and NE (H) in follicle rupture. The reduced response with R44E10-Gal4 labeling than R47A04-Gal4 is likely because it enables the isolation of slightly early stage-14 egg chambers. All conditions have three replicates except 0 and 20 μM OA, which have five replicates. (TIF) [file pgen.1005604.s005.tif]

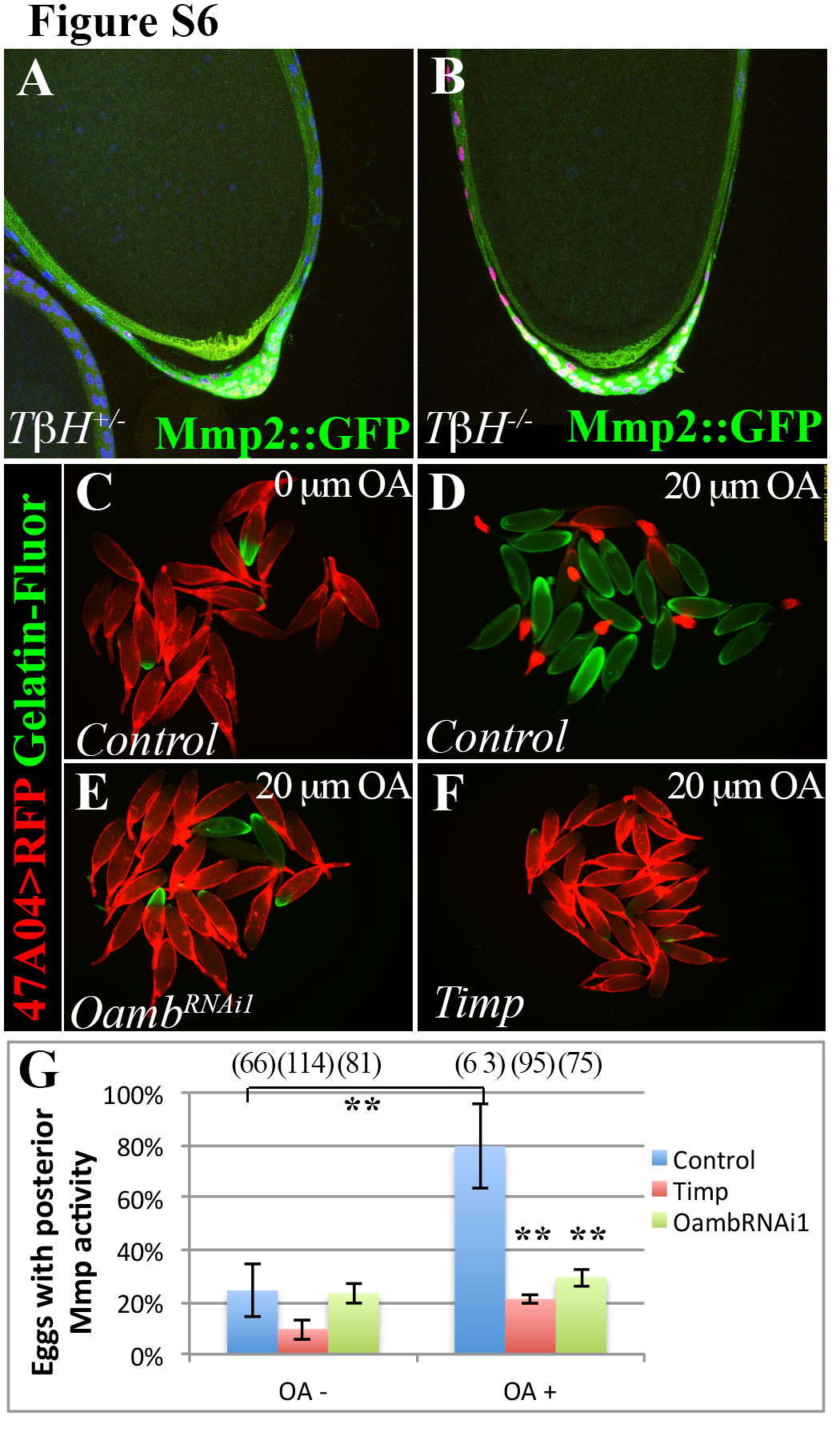

Supplement: S6 Fig — (A-B) Mmp2::GFP is expressed normally in posterior follicle cells of control (A) and TβH mutant (B) follicles. (C-F) Gelatinase activity in mature follicles after three-hour cultures without (C) or with (D-F) 20 μM of OA. Mature follicles were from control females (C-D) and females with 47A04-Gal4 driving Oamb RNAi1 (E) and Timp (F) expression. (G) Quantification of gelatinase activity from (C-F). ** P<0.01. All conditions have three replicates. (TIF) [file pgen.1005604.s006.tif]

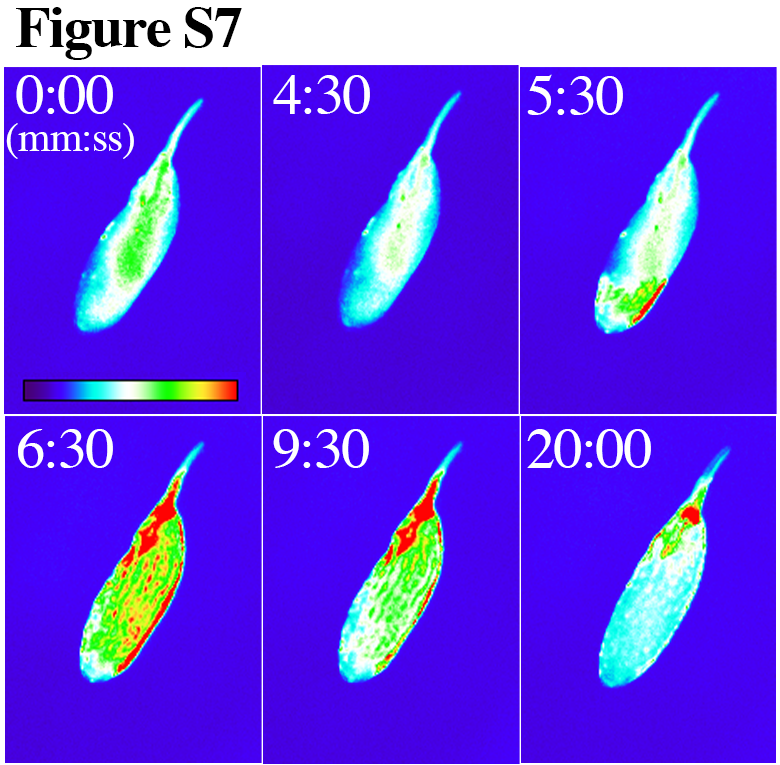

Supplement: S7 Fig — Ca2+ flux detected by R47A04-Gal4 driving UAS-GCaMP5G in mature follicle cells. The zero time point is 15 second before OA administration. The signal intensity was maximum around 6:30 (mm:ss). Fluorescence intensities are presented using a false-color scale, shown in the first panel. (TIF) [file pgen.1005604.s007.tif]

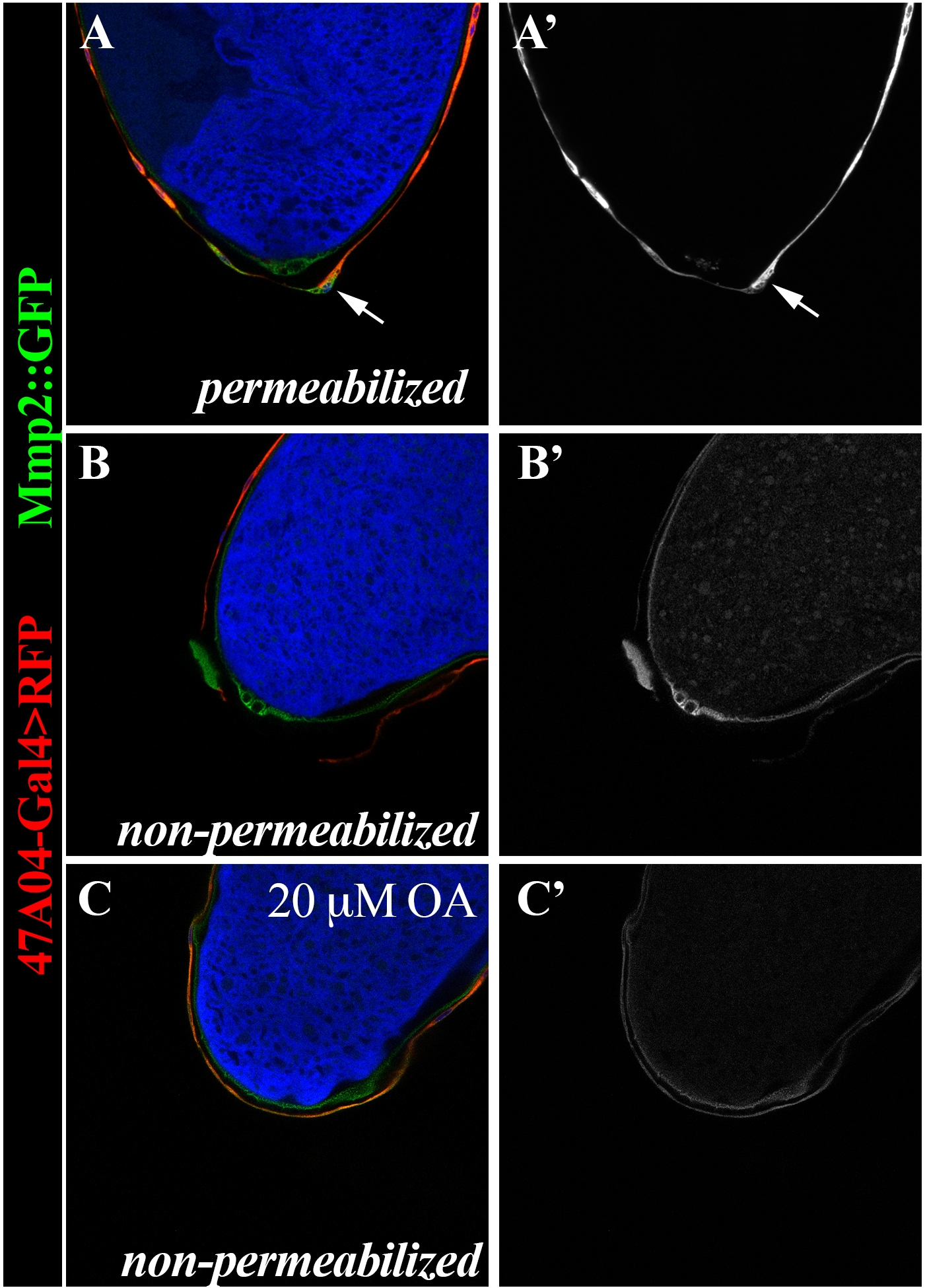

Supplement: S8 Fig — (A-A’) GFP antibody is applied after the fixation to permeabilize the cell membrane. Mmp2::GFP is detected in posterior follicle cells. (B-C’) GFP antibody is applied before the fixation to label the extracellular Mmp2::GFP. Mmp2::GFP is not detected in posterior follicle cells without (B-B’) and with (C-C’) OA stimulation. Together with the fact that Mmp2::GFP homozygous females are lethal, this result indicate that Mmp2::GFP fusion proteins are trapped inside the cell. (TIF) [file pgen.1005604.s008.tif]
