# Supplementary material for: A Follicle Rupture Assay Reveals an Essential Role for Follicular Adrenergic Signaling in Drosophila Ovulation
Source: PLoS Genet. 2015 Oct 16;11(10):e1005604. doi: 10.1371/journal.pgen.1005604 (PMC4608792; doi:10.1371/journal.pgen.1005604)
Supplement: S1 Table — (DOCX) [file pgen.1005604.s011.docx]

**Table S1. The analysis of kinetics of *ex vivo* follicle rupture**

| **Experiments** | **Total follicles for recording^f^** | **total ruptured during recording** | **# follicle for analysis** | **Starting time^d^ (min)** | **Finishing time^e^ (min)** | **Rupture time (min)** |
| --- | --- | --- | --- | --- | --- | --- |
| Movie 1^c^ | 20 | 7^a^ | 1 | 9.75 | 28.75 | 19.00 |
|  |  |  | 2 | 11.00 | 25.75 | 14.75 |
|  |  |  | 3 | 14.33 | 27.58 | 13.25 |
|  |  |  | 4 | 10.50 | 27.92 | 17.42 |
|  |  |  | 5 | 9.17 | 25.00 | 15.83 |
|  |  |  | 6 | 9.83 | 34.33 | 24.50 |
| Movie 2 | 8 | 4^a^ | 7 | 15.83 | 25.00 | 9.17 |
|  |  |  | 8 | 13.25 | 27.08 | 13.83 |
|  |  |  | 9 | 13.00 | 25.75 | 12.75 |
| Movie 3 | 15 | 8^b^ | 10 | 3.83 | 10.50 | 6.67 |
|  |  |  | 11 | 6.75 | 22.17 | 15.42 |
|  |  |  | 12 | 9.25 | 19.67 | 10.42 |
|  |  |  | 13 | 11.33 | 18.83 | 7.50 |
|  |  |  | 14 | 25.08 | 33.83 | 8.75 |
|  |  |  | 15 | 7.58 | 15.08 | 7.50 |
| **Average** |  |  |  |  |  | **13.12** |
| **Stdev** |  |  |  |  |  | **4.97** |

1. One follicles initiate rupture before recording.
2. Two follicles initiate rupture before recording.
3. The same as Movie S1.
4. Defined as the time frame when 5% of posterior tip without follicle-cell cover.
5. Defined as the time frame when more than 95% of oocytes without follicle-cell cover.
6. Follicles were isolated from five females for each experiment.
